# Oromandibular Dystonia as a Side Effect of Methotrexate

**DOI:** 10.7759/cureus.47248

**Published:** 2023-10-18

**Authors:** Diana Oliveira, David Moura, Sofia Azevedo, Bruno Guimarães, Sofia Toste

**Affiliations:** 1 Physical Medicine and Rehabilitation, Centro Hospitalar de Entre Douro e Vouga, Santa Maria da Feira, PRT

**Keywords:** methotrexate, adult-onset dystonia, meige’s syndrome, botulinum toxin, side effects

## Abstract

Oromandibular dystonia is a focal dystonia characterized by involuntary movements of the jaw, oropharynx, lips, and tongue. The diagnosis of oromandibular dystonia is clinical and can be complex. For effective treatment, it is essential to understand its underlying etiology. A 70-year-old man was referred to our center with a diagnosis of Meige’s syndrome, which had been present for five and a half years, for receiving botulinum toxin-A (BoNT-A) injections. Upon physical examination, he exhibited oromandibular dystonia, with a score of 177 points on the Oromandibular Dystonia Rating Scale (OMDRS). He had a history of taking methotrexate for six years, as he was diagnosed with psoriatic arthritis during that time. The possibility of methotrexate-induced dystonia was considered. A switch from methotrexate to sulfasalazine was initiated. Subsequently, the patient showed progressive improvement in his symptoms, as reflected by an OMDRS score of 103 points. After eight weeks, the medical team decided to supplement the treatment with BoNT-A injections, resulting in an OMDRS score of 75. While there is currently no definitive evidence linking the use of methotrexate to the development of dystonia, it is advisable to consider oromandibular dystonia as a potential side effect of methotrexate until more robust evidence becomes available.

## Introduction

Dystonia is characterized by repetitive, involuntary muscle contractions that result in abnormal movements. These contractions are often exacerbated by stress and fatigue. Dystonia can be classified based on its clinical characteristics, including age of onset, body distribution, temporal pattern, and associated features. Additionally, it can be categorized according to its etiology, which may be congenital or acquired (vascular, infectious, toxin-induced, medication-induced, autoimmune/inflammatory, metabolic, or neoplasms) [1,2].

Oromandibular dystonia is a focal dystonia characterized by involuntary movements of the jaw, oropharynx, lips, and tongue, which can impact speech and eating. It may present as jaw opening, jaw closing, jaw deviation, jaw protrusion, lingual dystonia, lip pursing, or chewing [1]. These movements can be rhythmic or tremor-like. The diagnosis of oromandibular dystonia is clinical and can be complex, as it can present in various ways and with varying degrees of severity. Treatment options range from addressing the underlying cause to using medication, physiotherapy, botulinum toxin injections, and afferent muscle block [3].

Blepharospasm is characterized by involuntary and spasmodic contractions of the periorbital muscles, leading to repetitive and uncontrolled closure of the eyelids [4].

Meige’s syndrome is characterized by oromandibular dystonia in association with blepharospasm and primarily affects women between the ages of 30 and 70. Its etiology is considered idiopathic, although there are hypotheses that link dopaminergic and cholinergic hyperactivity to the development of this syndrome. While there is no cure, there are drugs available to relieve symptoms, such as anticholinergics, antipsychotics, and GABA receptor agonists [4].

In light of the descriptions provided, it is evident that Meige’s syndrome, blepharospasm, and oromandibular dystonia are different conditions.

The objective of this clinical case is to emphasize the significance of a thorough clinical history in the differential diagnosis of dystonia and its impact on the quality of life of affected patients.

## Case presentation

A 70-year-old man was referred to our center for botulinum toxin-A (BoNT-A) injections as part of the management of Meige’s syndrome, which had been evolving for five and a half years.

In the patient’s medical history, he reported a progressive worsening of involuntary facial movements, which were exacerbated by stress and primarily affected the lower half of his face. During the physical examination, he exhibited oromandibular dystonia when initiating speech, but he was asymptomatic at rest (Video 1).

**Video 1:** Oromandibular dystonia.

Blood count, kidney and liver function, electrolyte panel, thyroid function, and glucose levels were within normal ranges. Cranioencephalic magnetic resonance imaging revealed no abnormalities.

Initially, the patient was treated with risperidone 4 mg, which resulted in moderate improvement. However, he had to discontinue the medication due to adverse effects, including increased anxiety, irritability, insomnia, and dizziness. Faced with a significant worsening of symptoms, it was decided to start biperiden 4 mg, but the patient could not tolerate it due to cognitive changes.

As part of his relevant medical history, he was diagnosed with psoriatic arthritis six years ago and had been taking methotrexate at a dose of 15 mg per week since the diagnosis.

The patient scored 177 points on the Oromandibular Dystonia Rating Scale (OMDRS) [5]. This scale assesses disease severity, psychosocial functioning, disability, quality of life, and the need for therapeutic changes [5].

Based on the patient’s clinical history, and the absence of blepharospasm, a delayed dystonia related to methotrexate intake was considered. After consulting with Rheumatology, it was decided to switch from methotrexate 15 mg/week to sulfasalazine 500 mg/day.

After three months, the patient showed significant improvement in his symptoms and achieved a score of 103 points on the OMDRS. Despite this improvement, the medical team decided to proceed with BoNT-A injection therapy.

**Figure 1:**
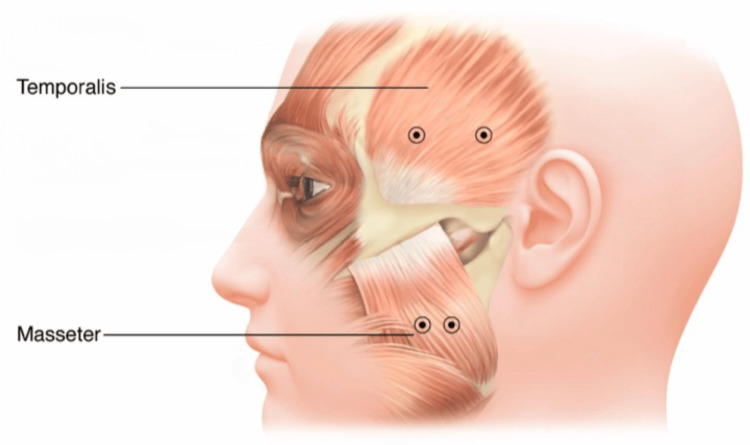
Botulinum toxin injection points in the masseter and temporal muscles. Figure adapted from Hassell et al. [6]. Note: This image was used with permission from the publisher. Proper attribution has been provided in accordance with the Creative Commons BY-NC 4.0 license, which allows non-commercial use with proper attribution.

OnabotulinumtoxinA was injected into the following muscles: masseter (20 U on the right and 20 U on the left) and temporal (20 U on the right and 20 U on the left) muscles (Figure 1).

Speech therapy rehabilitation was also recommended. The patient was re-evaluated after eight weeks and continued to experience progressive improvement in his oromandibular dystonia, with a score of 75 on the OMDRS. Despite the medication switch, the psoriatic arthritis remained well-controlled.

## Discussion

This patient presented with orofacial dystonia, which is one of the characteristic features of Meige’s syndrome. However, Meige’s syndrome is characterized by orofacial dystonia in association with blepharospasm [4]. The absence of blepharospasm raised doubts about the previously established diagnosis.

It is crucial to gather a comprehensive clinical history and conduct a thorough objective examination before proceeding with any treatment, regardless of the pre-existing diagnoses attributed to the patient.

Upon reviewing the patient’s medical history, there was a six-month gap between the initiation of methotrexate and the onset of dystonia, which complicates the diagnosis and makes it challenging to establish a direct link between the drug and the adverse effect. Nevertheless, this timeframe is insufficient to rule out this possibility, as some adverse effects may only manifest several years after commencing the medication.

The clinical improvement observed over time and documented through the OMDRS following the discontinuation of methotrexate is consistent with the diagnosis of oromandibular dystonia as an adverse effect of methotrexate, rather than Meige’s syndrome.

A score of 5 on the Naranjo Adverse Drug Reaction Probability Scale indicates a probable relationship between methotrexate and the development of oromandibular dystonia [7].

Despite the significant improvement observed, dystonic movements were still noticeable and had some impact on the patient’s daily living activities. To alleviate the symptoms caused by oromandibular dystonia, the use of botulinum toxin is recommended [8]. Botulinum toxin is derived from the neurotoxin produced by *Clostridium botulinum* and functions by temporarily inhibiting the release of acetylcholine at the neuromuscular junction, thereby blocking nerve impulse conduction, leading to muscle relaxation and decreased spasm [8]. Therefore, it was decided to perform infiltration of the most affected muscles.

However, it is important to note that the effect of botulinum toxin decreases after eight weeks, returning to the baseline at 12 weeks [8], so the patient can expect to undergo injections every 12 weeks if residual dystonia symptoms persist, even after discontinuing methotrexate.

While there is currently no conclusive evidence establishing a direct link between methotrexate use and the development of oromandibular dystonia, there are case reports that raise questions about a possible association [9,10]. One such case, similar to the one presented here, detailed the emergence of oro-bucco-lingual dyskinesia two months after starting methotrexate, with significant improvement after discontinuation [9]. Another case described the development of oromandibular dystonia six months after initiating methotrexate, but the authors associated it with the patient’s history of facial injury and edentulosity. However, in this case, it should be considered that the patient had previously experienced edentulosity and facial injury, so the association with the introduction of methotrexate cannot be ruled out [10]. According to a phase IV clinical study, 0.01% of individuals taking methotrexate reported the development of tardive dyskinesias [11].

## Conclusions

This clinical case highlights the complexity of diagnosing and managing oromandibular dystonia. Thorough clinical history-taking and a comprehensive objective examination are essential in distinguishing between different types of dystonia and identifying potential contributing factors, such as medication-induced adverse effects. In this case, the temporal relationship between methotrexate initiation and the onset of dystonia raised suspicions about a possible association. It is not possible to predict whether the dystonia will continue to improve, despite drug withdrawal; however, the remaining symptoms can be managed with botulinum toxin injections and speech therapy.

While the direct link between methotrexate and oromandibular dystonia remains uncertain and requires further investigation, the case serves as a reminder that medication-induced adverse effects should always be considered in clinical practice. It underscores the significance of ongoing research and reporting of such cases to expand our understanding of potential associations and improve patient care. In essence, this case emphasizes the critical role of a multidisciplinary approach, thorough evaluation, and individualized treatment strategies in the management of dystonia, ultimately aiming to enhance the quality of life for affected patients.
